# Assessing Patients’ Preferences for Preventive Dental Care: A Discrete Choice Experiment

**DOI:** 10.7759/cureus.44028

**Published:** 2023-08-24

**Authors:** Lina Bahanan, Fatmh Bashkail, Afrah Alghamdi, Ahlaa Alhazmy

**Affiliations:** 1 Dental Public Health, King Abdulaziz University, Faculty of Dentistry, Jeddah, SAU; 2 General Dentist, King Abdulaziz University, Faculty of Dentistry, Jeddah, SAU; 3 Oral and Maxillofacial Rehabilitation, King Abdulaziz University, Faculty of Dentistry, Jeddah, SAU

**Keywords:** discrete choice experiment, primary prevention, dental visits, dental health services, preventive dentistry

## Abstract

Background

It is crucial to recognize patients’ behavioral responses to improve oral healthcare delivery. A discrete choice experiment (DCE) is typically used to measure service user preferences. The purpose of this study was to examine the preferences of the Saudi population regarding primary dental care using a DCE.

Materials and methods

An online DCE survey was distributed among adults aged 18 years and older living in Saudi Arabia. An orthogonal design was used to reduce the number of combinations while maintaining the main effects that reflect patient preferences. Respondents were presented with a reduced set of 10 cards (tasks), and each of the cards had two concepts (offers). Descriptive statistics were used to summarize the sample characteristics. The coefficients were calculated based on discrete choice results on the Qualtrics platform.

Results

A total of 532 participants completed the survey. Among patients seeking a preventive dental visit, the ability to get an appointment was rated the most important influence on their decision with a relative importance of 41%, followed by the dental care provider (29%) and the dental clinic in relation to the sector and the fees (20%). The least important attribute was the waiting time in minutes, with a relative importance of 10%.

Conclusion

The study findings revealed that patients make trade-offs when deciding to receive primary dental care. Policymakers can use the study’s findings to inform their decisions concerning dental clinic services. The study results suggest that it is possible to improve access to primary dental care by improving the accessibility of appointments.

## Introduction

Global health care systems aim to provide person-centered care [1]. A crucial step in improving healthcare delivery is recognizing patients’ behavioral responses, as they are often overlooked [2]. The preferences of patients have been found to be highly heterogeneous [3,4]. Understanding the preferences of patients may help provide better health care services [3,4].

Measurements of service user preferences are commonly conducted using discrete choice experiments (DCEs) [1]. DCEs have been commonly used to ascertain the preferences of healthcare service users, assign financial values to healthcare attributes, and anticipate the use of specific services [2,5-10]. An initial step in designing a DCE is to identify the attributes relevant to the research question, such as waiting times, costs, or service providers [11]. Following this, levels are assigned to these attributes. Respondents must be presented with relevant and realistic scenarios at each level [11].

The willingness to make trade-offs between such attributes can therefore be identified, such as preferring a dentist over a hygienist to perform scaling and polishing; however, service providers may be sacrificed for cost savings, whereas clinical outcomes may be forgone in favor of aesthetics [1]. DCE studies have shown that respondents tend to overlook one or more attributes, resulting in a biased willingness to pay estimates [12-14]. However, underlying preferences have been estimated, assuming that all the attributes were taken into account [13]. According to Sever et al. (2019), a discrete choice assessment revealed significant differences between patients’ preferences. Their findings revealed that the most valued attribute of dental care was the patient’s willingness to pay for a service, followed by the behavior of the dental staff [15].

The use of DCEs is an effective method for determining patients’ preferences [11]. Nevertheless, their application in the dental field is limited, and they have not yet been used in Saudi Arabia. The purpose of this study is to investigate the preferences of the people living in Saudi Arabia regarding primary dental care services. This study will provide insights for policymakers regarding the organization of dental care services.

## Materials and methods

Study sample

This cross-sectional study was conducted between September 2022 and February 2023. Participants were recruited using a non-probability snowball sampling technique. The inclusion criteria included participants living in Saudi Arabia and aged 18 years or older. Participants who lived outside Saudi Arabia or were younger than 18 years of age were excluded from the study. This study was reviewed and approved by the Institutional Review Board at King Abdulaziz University Faculty of Dentistry, Jeddah, Saudi Arabia (#090-9-22).

Selection of attributes and levels

A literature review was conducted to determine the relevant attributes and their levels [1,6,15]. After obtaining ethical approval, a focus group interview was conducted with six to ten individuals to determine additional attributes.

Questionnaire development

The survey is composed of two sections. The first section includes demographic questions on the participants’ gender, age, marital status, nationality, education, employment status, income, region, and dental insurance. The second section is composed of choice tasks along with an explanation of the attributes and their levels. Dental clinics from various regions were contacted to determine the range of dental cleaning costs. The attributes and their levels are shown in Table 1. 

**Table 1:** The attributes and their levels

Attributes	Levels
Ability to get an appointment	Ability to get an appointment within two-three weeks
	Ability to get an appointment within one-two months
	Ability to get an appointment after three months
Who performed the dental cleaning	Dentist
	Hygienist
	Either the dentist or hygienist
The dental clinic	Free government dental clinics
	Private dental clinics costing 95–250 SR
	Private dental clinics costing 251–450 SR
Waiting time in minutes	Five–15 minutes
	15–30 minutes
	> 30 minutes

The full factorial design of the DCE includes all 81 (3 × 3 × 3 × 3) combinations of the levels of the attributes. Using an orthogonal design, the number of combinations was reduced to prevent respondent fatigue while retaining the main effects of combinations reflecting patient preferences. Qualtrics was used to carry out the orthogonal experimental design. Each respondent was presented with a reduced set of 10 cards (tasks), each of which had two concepts (offerings) from which they had to choose one. Table 2 shows examples of the alternatives presented in the DCE.

**Table 2:** Examples of the alternatives presented in the DCE DCE: discrete choice experiment

Attributes	Alternative 1	Alternative 2	Alternative 3	
Ability to get an appointment	Ability to get an appointment after three months	Ability to get an appointment within one-two months	Ability to get an appointment within two-three weeks	
				Who performed the dental cleaning	Either dentist or hygienist	Dentist	Hygienist	
The dental clinic	Free government dental clinics	Private dental clinics costing 251–450 SR	Private dental clinics costing 251–450 SR					
				Waiting time in minutes	> 30 minutes	15–30 minutes	Five–15 minutes	

The survey was provided in English and Arabic through the Qualtrics platform. The survey was distributed online through social media. Descriptive statistics were used to summarize the sample characteristics. The Qualtrics platform calculated the coefficients (utilities) based on discrete choice results.

Sample size

A pilot study was conducted with 30 participants. According to Orme (2010), a sample size of 200 respondents is recommended for DCE research that requires analyzing sample segment differences, or 300 respondents if no segment differences were examined [16]. The total sample included 500 respondents to account for missing observations. The data were analyzed using IBM SPSS Statistics for Windows, Version 20.0 (Released 2011; IBM Corp., Armonk, New York, United States).

## Results

Of the 965 respondents, 532 completed the survey. Based on the inclusion and exclusion criteria, 512 were eligible. The data set includes 15,360 observations (512 respondents × 10 completed choice tasks × 3 choice task alternatives). The characteristics of the study population are presented in Table 3. Over two-thirds of the study sample were female, single, and aged 18-29. Most respondents reported having a Bachelor’s degree (62.7%). The majority of the study participants were Saudi (91.1%), and their monthly income was less than 10,000 SR (70.7%). About 69% of the participants did not have dental insurance. The sample was diverse in terms of regions (Table 3).

**Table 3:** Characteristics of the study population (n = 512) Some variables do not add up to the total because of missing data

Characteristics	Frequency (%)
Gender	
Male	187 (36.7)
Female	323 (63.3)
Age	
18–29	330 (64.6)
30–49	117 (22.9)
50 +	64 (12.5)
Marital status	
Single	318 (62.2)
Married	181 (35.4)
Divorced	10 (2.0)
Widowed	2 (0.4)
Nationality	
Saudi	468 (91.1)
Non-Saudi	33 (6.4)
Level of education	
High school or less	101 (19.7)
Diploma degree	44 (8.6)
Bachelor’s degree	321 (62.7)
Postgraduate degree	46 (9.0)
Employment status	
Unemployed	80 (15.6)
Retired	39 (7.6)
Employee: government sector	77 (15.0)
Employee: private sector	88 (17.2)
Student	228 (44.5)
Income per month	
Less than 4,000 SAR	238 (47.0)
4,000–10,000 SAR	120 (23.7)
10,001–20,000 SAR	103 (20.4)
More than 20,000 SAR	45 (8.9)
Region	
Western	119 (23.2)
Northern	62 (12.1)
Eastern	118 (23.1)
Southern	55 (10.7)
Central	158 (30.9)
Dental insurance	
Yes	159 (31.1)
No	353 (68.9)

**Figure 1:**
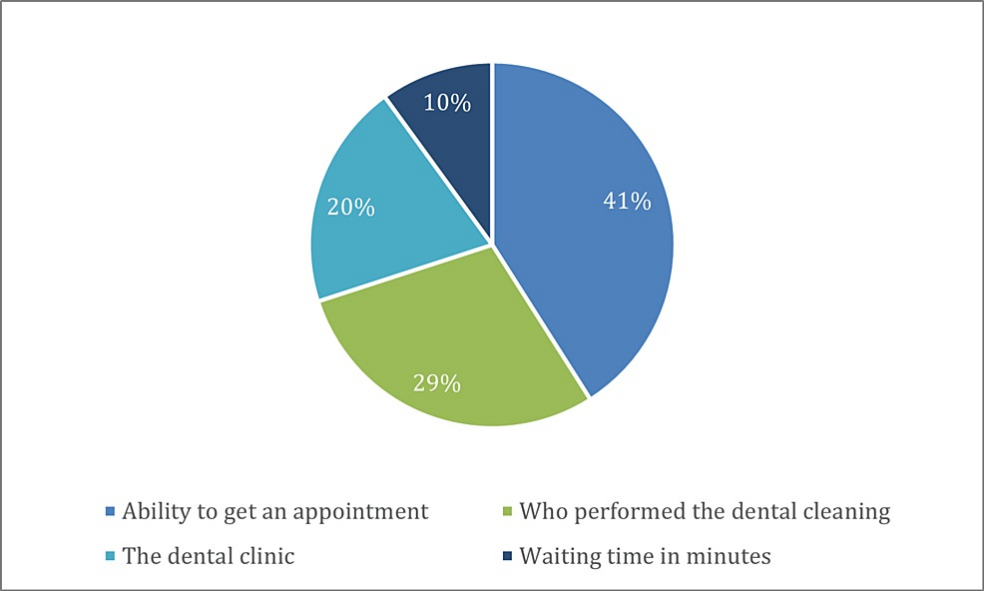
Discrete choice experiment: relative importance of the four attributes

Based on the evaluated attributes, the “Ability to get an appointment” was ranked as the most important influence on patients seeking dental cleaning, with a relative importance of 41%, followed by “Who performed the dental cleaning,” with a relative importance of 29%, the “Dental clinic in relation to the dental sector and the fees,” which had a relative importance of 20%, and, lastly, the “Waiting time in minutes,” which was ranked as the least influential, with a relative importance of 10% (Figure 1).

**Figure 2:**
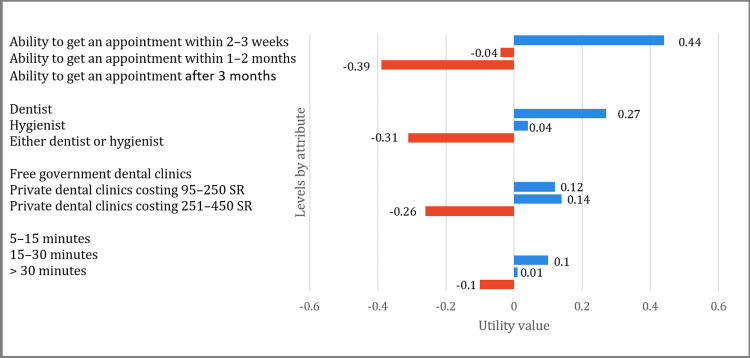
Discrete choice experiment: utility values of each level of the attributes

According to the utility values, patients prefer to be given an appointment within two to three weeks rather than months, and one to two months was preferred over three months. In line with expectations, the patients strongly preferred to have a dentist perform the procedure instead of a hygienist or provider not chosen by them. Moreover, no significant difference was observed between the private dental clinics charging 95-250 SR and the free governmental dental clinics. However, there was a strong disinclination among the patients toward private dental clinics that charge 251-450 SR. Lastly, they were content with a waiting time of five-15 minutes and somewhat content with a waiting time of 15-30 minutes when compared to a waiting time greater than 30 minutes (Figure 2).

## Discussion

This study aimed to investigate the preferences of people living in Saudi Arabia regarding preventive dental care services. Our findings revealed that the “Ability to get an appointment” greatly influences patients’ willingness to seek dental cleanings, which has the highest relative importance. Patients were willing to trade other attributes, such as the dental care provider, dental clinic sector, cost, and waiting time, to get an appointment at the earliest possible time. These findings coincide with those of the study by Kim et al., who examined the reasons behind patients’ choice of dentist at the University of Iowa College of Dentistry [17]. The authors found that a reasonable waiting time with regard to getting an appointment was highly ranked. On the other hand, a study in New Zealand by Gray et al. examined what motivates patients to seek dental care and the barriers they face [18]. They found that appointment availability was one of the least important factors [18]. This might be due to the tendency of the study population to request appointments for urgent complaints rather than for regular dental check-ups or preventative treatment. According to El Bcheraoui et al., only 11.5% of Saudis aged 15 or older had had a routine dental check-up within the last year [19].

Our findings suggest that patients prefer dentists over dental hygienists or dental providers not chosen by them. These findings are consistent with those of Boyers et al., who used a DCE to elicit preferences for scale and polish (SP) and personalized oral hygiene advice (OHA) services in the UK [1]. They found that many UK patients preferred to receive some of these services (personalized OHA and a 12-month SP) from a dentist rather than a hygienist [1]. This might be due to the fact that dentists are better able to identify other dental health problems and are more experienced at performing these procedures than hygienists. Consequently, the dental services provided by a dentist are more expensive than those offered by a hygienist.

Regarding the dental clinics attribute, the utility value of the free governmental clinics is somewhat the same as that of those in the private sector that charge 95-250 SR. However, private sector charges exceeding 250 SR have a high negative utility value, suggesting that many patients are price-sensitive. Nevertheless, the dental clinic attribute has a lower relative importance than the dental care provider attribute. Therefore, it would appear that patients are willing to trade the dental clinic with regard to sector and fee for the ability to choose which dentist provides the service.

Our findings showed that patients assign less importance to waiting times in dental offices. A study by Sever et al. also found waiting times to be the least important attribute [4]. Most of the participants would be willing to pay an extra 38 HRK (20 SR) to decrease the waiting time from 20 minutes to five minutes [4]. In addition, older and/or more highly educated patients tended to be more irritated by a long waiting time (45 minutes), which is expected as elderly patients often require special care [4]. It is therefore essential to have a convenient appointment system.

This study is considered the first to investigate the preferences of people living in Saudi Arabia for primary dental care services using a DCE. DCE studies provide a deeper understanding of patients' needs and expectations and allow for more patient-centered care. Thus, insurance policies and regulations can be changed in a way that benefits the population at large. However, this study has some limitations. It is a cross-sectional study, which is considered to rank low in terms of the strength of its evidence. Considering this is a pilot study, more studies need to be conducted on a larger number of populations to allow for generalizability. In our study, we used an online survey, and such a method is frequently criticized for two methodological flaws: the inability to describe how the respondents were selected and the possibility that respondents may be biased when selecting themselves. This study provides valuable insights into people's preferences regarding primary dental care services in Saudi Arabia. Patients' decisions are influenced most by appointment availability, which highlights the need to improve access to dental care by making appointments readily available. This could be achieved by increasing the number of dental clinics or expanding the operating hours of existing clinics to accommodate more patients. Additionally, the study found that patients place a high value on dental care providers, followed by dental clinics in terms of sector and fees. This suggests that policymakers should focus on retaining qualified dental professionals to meet patient expectations. Additionally, efforts should be made to ensure that dental services are affordable for all patients, regardless of their socioeconomic status. Health policymakers can use the findings of this study to develop strategies for improving the delivery of primary dental care services and ensuring access to high-quality, affordable dental care for all patients.

## Conclusions

Obtaining a convenient dental cleaning appointment takes precedence over other attributes, with dental care providers coming in a distant second, followed by the dental sector and fees, and, finally, the waiting time. In light of the findings of this study, dental clinics may be able to focus on those attributes that patients value the most.
